# Azithromycin and Sildenafil May Have Protective Effects on Retinal Ganglion Cells via Different Pathways: Study in a Rodent Microbead Model

**DOI:** 10.3390/ph16040486

**Published:** 2023-03-24

**Authors:** Tal Corina Sela, Alon Zahavi, Moran Friedman-Gohas, Shirel Weiss, Amir Sternfeld, Astrid Ilguisonis, Danielle Badash, Noa Geffen, Ron Ofri, Yaniv BarKana, Nitza Goldenberg-Cohen

**Affiliations:** 1 Clalit Health Services, Tel Aviv 6209804, Israel; 2 Sackler Faculty of Medicine, Tel Aviv University, Tel Aviv 6997801, Israel; 3 Department of Ophthalmology, Rabin Medical Center—Beilinson Hospital, Petach Tikva 4941492, Israel; 4 Laboratory of Eye Research, Felsenstein Medical Research Center, Petach Tikva 4941492, Israel; 5 Krieger Eye Research Laboratory, Rappaport Faculty of Medicine, Technion—Israel Institute of Technology, Haifa 3200003, Israel; 6 Koret School of Veterinary Medicine, Hebrew University of Jerusalem, Rehovot 7610001, Israel; 7 The Glaucoma Innovations and Research Laboratory, The Sam Rothberg Glaucoma Center, Sheba Medical Center, Tel Hashomer 5262000, Israel; 8 Department of Ophthalmology, Bnai Zion Medical Center, Haifa 3339419, Israel; 9 Bruce and Ruth Faculty of Medicine, Technion, Technion—Israel Institute of Technology, Haifa 3200003, Israel

**Keywords:** glaucoma, microbeads, neuroprotection, sildenafil, azithromycin

## Abstract

Decreased blood flow to the optic nerve (ON) and neuroinflammation are suggested to play an important role in the pathophysiology of glaucoma. This study investigated the potential neuroprotective effect of azithromycin, an anti-inflammatory macrolide, and sildenafil, a selective phosphodiesterase-5 inhibitor, on retinal ganglion cell survival in a glaucoma model, which was induced by microbead injection into the right anterior chamber of 50 wild-type (WT) and 30 transgenic toll-like receptor 4 knockout (TLR4KO) mice. Treatment groups included intraperitoneal azithromycin 0.1 mL (1 mg/0.1 mL), intravitreal sildenafil 3 µL, or intraperitoneal sildenafil 0.1 mL (0.24 μg/3 µL). Left eyes served as controls. Microbead injection increased intraocular pressure (IOP), which peaked on day 7 in all groups and on day 14 in azithromycin-treated mice. Furthermore, the retinas and ON of microbead-injected eyes showed a trend of increased expression of inflammatory- and apoptosis-related genes, mainly in WT and to a lesser extent in TLR4KO mice. Azithromycin reduced the BAX/BCL2 ratio, TGFβ, and TNFα levels in the ON and CD45 expression in WT retina. Sildenafil activated TNFα-mediated pathways. Both azithromycin and sildenafil exerted a neuroprotective effect in WT and TLR4KO mice with microbead-induced glaucoma, albeit via different pathways, without affecting IOP. The relatively low apoptotic effect observed in microbead-injected TLR4KO mice suggests a role of inflammation in glaucomatous damage.

## 1. Introduction

Glaucoma is a progressive, sight-threatening, neurodegenerative ocular disease characterized by early damage to and loss of retinal ganglion cells (RGCs) and their axons [1,2]. RGC death occurs by apoptotic mechanisms [1,3]. The pathophysiology underlying glaucoma is still only partially understood, so medical treatment is targeted at reducing the intraocular pressure (IOP) [1], by either decreasing the production of aqueous humour or increasing its drainage [1,2]. However, the benefit of these treatments is limited.

One of the main hypotheses of the biological basis of glaucoma suggests that decreased blood flow to the optic nerve may contribute to the glaucomatous damage [2]. Sildenafil (Viagra^®^) is a selective phosphodiesterase-5 (PDE-5) inhibitor that causes vasodilatation. It has been in wide use for over two decades, mainly for the treatment of erectile dysfunction but also for pulmonary arterial hypertension and other clinical conditions [4]. Sildenafil induces smooth muscle relaxation by decreasing PDE-5 activity, resulting in elevated cyclic guanosine monophosphate (c-GMP) concentration, which potentiates nitric oxide vasodilatory activity. In recent years, evidence has accumulated that via these c-GMP-mediated pathways, sildenafil may exert a neuroprotective effect in various conditions associated with restricted blood flow [3,5,6,7,8,9]. In the retina, PDE-5 plays a role in regulating neuronal survival [4,7]. Thus, sildenafil might serve as a neuroprotector of the RGCs and optic nerve in glaucoma. Notwithstanding, as previously reported, systematic treatment with sildenafil can elicit anterior ischemic optic neuropathy (AION) [4,10,11]. Intravitreal administration of sildenafil could potentially reduce this risk with no reported ocular or systemic toxicity [7].

Tumor necrosis factor alpha (TNFα)–mediated neuroinflammation is another proposed mechanism for glaucoma progression [1,12]. In an earlier study, our group found evidence of an inflammatory response following induction of glaucoma in mice [13].

Azithromycin (AZ) is a commonly prescribed macrolide antibiotic with an anti-inflammatory effect [14,15]. It reduces chemokine production by neutrophils and modifies oxidative burst, diminishes prostaglandin synthesis by the inhibition of COX enzymes, decreases TNFα levels, and suppresses prolonged inflammation [14,15]. In recent years, it has been shown to provide a neuroprotective effect in a neonatal rat model of hypoxic-ischemic brain injury [16], to prevent RGC death in acute retinal ischemic injury in rats [17], and to provide neuroprotection in rats following transient focal cerebral ischemia, an effect that was accompanied by elevated STAT3 phosphorylation [18]. Hence, it may serve to diminish the possible destructive inflammatory component in glaucoma pathophysiology.

The objective of the present study was to investigate the possible neuroprotective effect of AZ and sildenafil on RGCs and optic nerve damage in an experimental microbead glaucoma model. Toll-like receptor 4 knockout (TLR4KO) mice were used in order to isolate the inflammatory effect. Better understanding of the mechanisms involved in glaucomatous damage and the potential to inhibit specific targets may lead to the development of new topical medications directed at the inflammatory reaction and not the IOP.

## 2. Results

### 2.1. IOP Measurements

**Figure 1:**
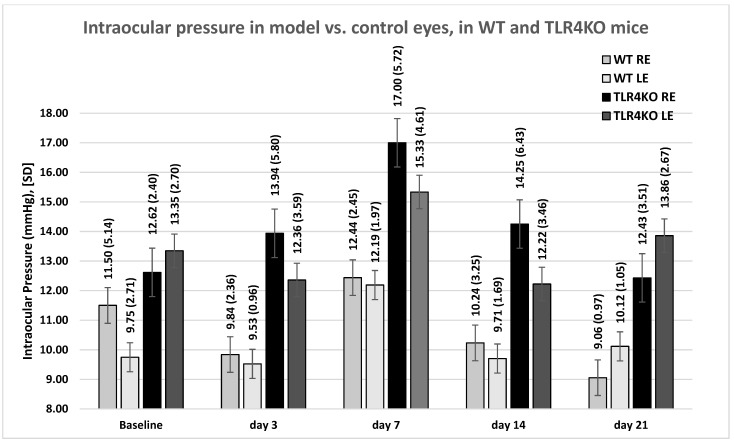
IOP measurements. Mean (SD) IOP values in the model (right) versus control (left) eyes of WT and TLR4KO mice by day of measurement. WT, wild type; TLR4KO, toll-like receptor 4 knockout; RE, right eyes; LE, left eyes.

**Figure 2:**
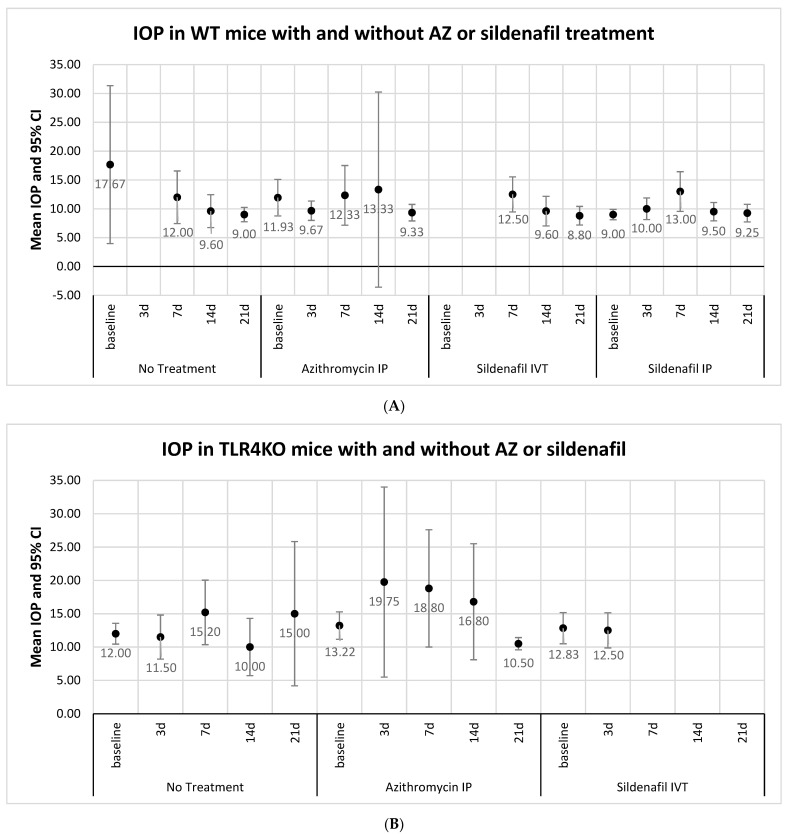
(**A**) Comparison of IOP measurements between treated and untreated groups: WT mice. Analysis of eyes injected with microbeads and either untreated (*n* = 8) or treated with intraperitoneal AZ (*n* = 20), intravitreal sildenafil (*n* = 5), or intraperitoneal sildenafil (*n* = 17). The results shown in the figure are based on between 3 and 15 eyes for each measurement. IOP decreased in systemic untreated eyes during follow-up. IOP was variable in AZ-treated eyes, peaking at 2 weeks. In sildenafil-treated eyes, IOP peaked on day 7 and then decreased. IOP, intraocular pressure; IP, intraperitoneal; IVT, intravitreal. (**B**) Comparison of IOP measurements between treated and untreated groups: TLR4KO mice. Analysis of eyes injected with microbeads and either untreated (*n* = 11) or treated with intraperitoneal AZ (*n* = 9). The results of 3–15 eyes for each measurement are shown. IOP increased in AZ-treated eyes on days 3, 7, and 14.

IOP was measured in right (microbead-induced glaucoma) eyes and left (control) eyes. In both WT and TLR4KO mice, baseline IOP (10.69 ± 4.25 mmHg and 12.85 ± 2.54 mmHg, respectively) rose and peaked on day 7 (12.31 ± 2.19 mmHg and 16.21 ± 5.15 mmHg, respectively). Looking at all right (study) eyes together, including eyes receiving only microbead injection and eyes that were also treated with AZ or sildenafil, a tendency (without statistical significance) towards higher IOP with greater IOP variability compared with the control eyes was observed during the follow-up period (Figure 1). Additionally, IOP was higher in glaucomatous eyes of TLR4KO mice than of WT mice (Figure 1), but this trend was also observed in control eyes. Treatment with either AZ or sildenafil did not result in a significant change of IOP compared with no treatment among eyes with microbead-induced glaucoma (Figure 2A,B). To summarize, IOP was mildly elevated in study eyes compared with the control eyes. Within intervened eyes, treatment with either AZ or sildenafil did not affect IOP.

### 2.2. Histology

**Figure 3:**
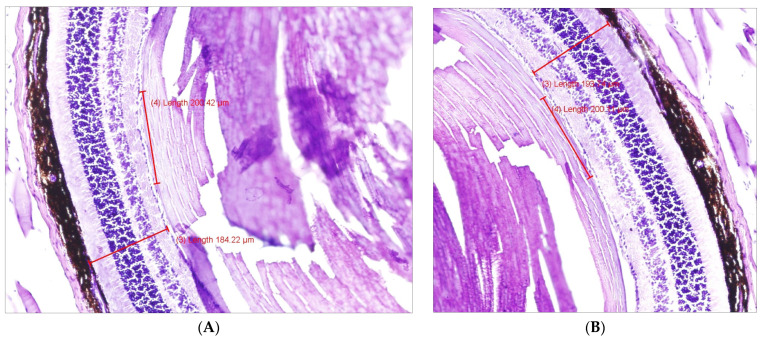
H&E staining showing the retinal thickness in WT mice. (**A**) microbead-injected eyes showing a retinal thickness of 184.22 µm. (**B**) control eyes showing a retinal thickness of 193.74 µm. H&E, hematoxylin and eosin; WT, wild type.

Histological analysis was performed after euthanasia on day 21 in WT and TLR4KO mice. Right eyes in each treatment group were compared with their left controls. In the WT mice that received microbead injection and no medicinal treatment, mean retinal thickness and mean RGC count were significantly lower in the (right) eyes with microbead-induced glaucoma than in the (left) control eyes (retinal thickness: 183.27 µm vs. 198.34 µm, *p* = 0.009; RGC count in a 200 µm section: 20.37 vs. 22.41, *p* = 0.003). Figure 3 shows an example of an H&E-stained retinal section in the study (Figure 3A) compared with control (Figure 3B) eyes. Similar findings were observed upon injection of AZ into the right eyes, with favorable retinal thickness (*p* = 0.002) and RGC count (*p* < 0.001) in left eyes. However, on injection of sildenafil, the difference from controls was smaller and did not achieve statistical significance (Table 1A). In the model (microbeads only) TLR4KO mice, there was no significant difference in RGC count between the right and left eyes (21.83 and 18.6, respectively, *p* = 0.171).

Right eyes from AZ- or sildenafil-treated groups were also compared with those of the microbead-only group. In the WT mice, injection of either AZ or sildenafil was associated with the preservation of retinal thickness and RGC count compared with no treatment. The mean retinal thickness was 183.27 µm in untreated model eyes, 189.61 µm in AZ-treated eyes, and 192.55 µm in sildenafil-treated eyes. The difference in retinal thickness was statistically significant for sildenafil treatment vs. no treatment (*p* = 0.037). The corresponding mean RGC counts were 20.37, 21.87, and 21.12. The difference in RGC count was significant for AZ treatment vs. no treatment (*p* = 0.017) (Table 1B).

In conclusion, in WT mice, microbead-injection led to retinal thinning with RGC death. AZ and sildenafil treatments both showed neuroprotective effects.

### 2.3. Molecular Analysis

Molecular analysis was performed following euthanasia in WT and TLR4KO mice on day 3 after induction of the model.

#### 2.3.1. Glaucoma Microbead Model: WT Mice

**Figure 4:**
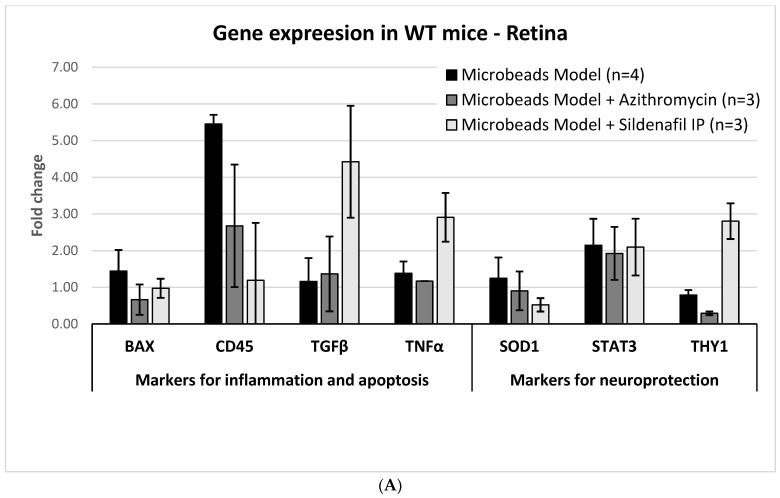

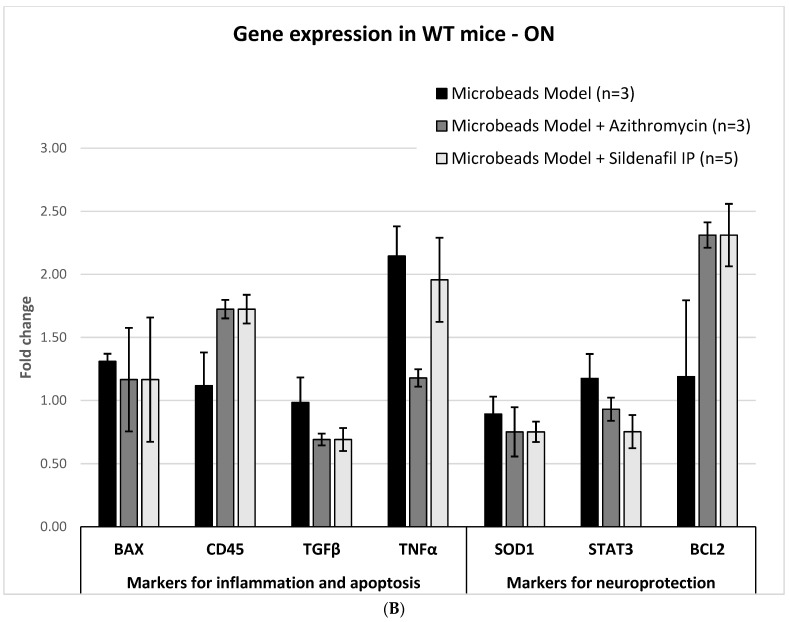

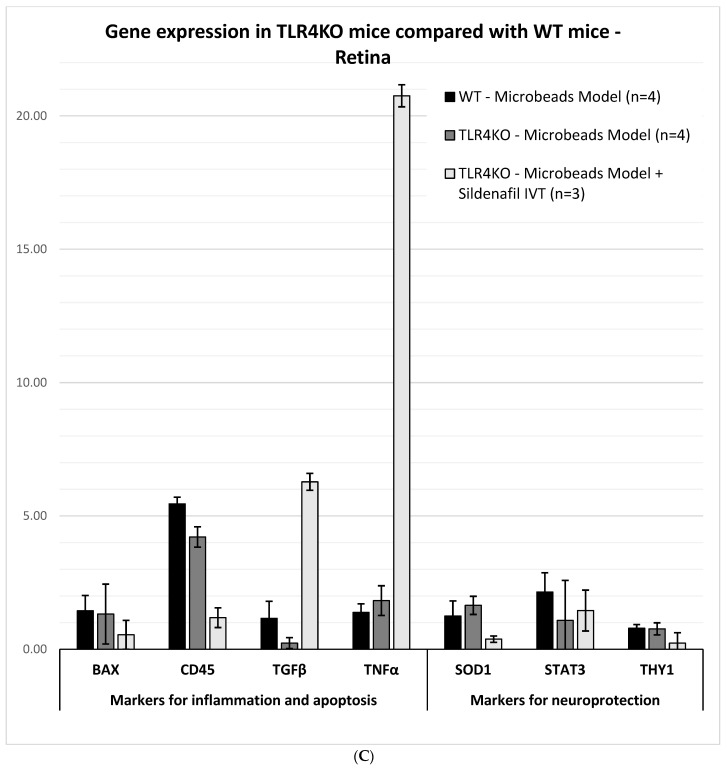
(**A**) Fold changes in gene expression (mean and SD) by treatment group: WT mice, retina. BAX increased in glaucoma-induced (model) eyes and decreased with AZ or sildenafil treatment. CD45 markedly increased in model eyes, and less or not at all with AZ or sildenafil treatment, respectively. TGFβ and TNFα remained at baseline levels in untreated and AZ-treated eyes, and increased in sildenafil-treated eyes. There was no effect on elevated STAT3 in all groups. SOD1 mildly decreased with AZ treatment and further decreased with sildenafil treatment. THY1 slightly decreased in model eyes, markedly decreased with AZ treatment, and increased with sildenafil treatment. (**B**) Fold changes in gene expression (mean and SD) by treatment group: WT mice, optic nerve. BAX increased in glaucoma-induced eyes with no change with treatment. CD45 remained at baseline in model eyes and increased with AZ or sildenafil treatment. TGFβ remained at baseline in model eyes and decreased with AZ or sildenafil treatment. TNFα was elevated in model eyes and normalized with AZ but not with sildenafil treatment. SOD1 mildly decreased with AZ or sildenafil treatment. STAT3 mildly increased in model eyes, normalized with AZ treatment, and further decreased with sildenafil treatment. There was a marked elevation in BCL2 with AZ and sildenafil treatment. (**C**) Fold changes in gene expression (mean and SD) by treatment group: TLR4KO mice, retina, including a comparison to retinas of model eyes in WT mice. Sildenafil reduced the levels of BAX, CD45, SOD1, and THY1. There was a marked elevation in TGFβ and an extreme elevation in TNFα with sildenafil treatment. SD, standard deviation; WT, wild type; IP, intraperitoneal; ON, optic nerve; TLR4KO, toll-like receptor 4 knockout; IVT, intravitreal.

In the WT mice, molecular analysis of the right retina with microbead-induced glaucoma revealed increased expression of the following genes (mean ± SD): BAX 1.44 ± 0.58 fold; TNFα, 1.38 ± 0.32 fold; CD45, 5.45 ± 0.25 fold; STAT3, 2.15 ± 0.72 fold. In the optic nerve, BAX and CD45 were mildly elevated, but TNFα increased considerably (2.15 ± 0.23 fold). The BAX/BCL2 ratio was 1.1. TGFβ levels did not change, either in the retina or in the optic nerve (Figure 4A,B).

#### 2.3.2. AZ Treatment: WT Mice

Compared with untreated microbead-injected eyes, the retinas of model eyes treated with AZ showed a decrease in BAX (mean ± SD) levels (1.44 ± 0.58 fold vs. 0.66 ± 0.41 fold) and in CD45 (5.45 ± 0.25 fold and 2.68 ± 1.67 fold) (Figure 4A). In the optic nerve, AZ treatment was associated with a decreased expression of the inflammatory markers TGFβ (0.69 ± 0.05 fold) and TNFα (1.18 ± 0.07 fold). In the ON, the levels of CD45 increased (1.72 ± 0.07 fold), BAX remained stable, and BCL2 markedly increased (2.31 ± 0.1 fold versus 1.19 ± 0.6 fold in the untreated model ONs), leading to a decrease from 1.1 to 0.5 in the BAX/BCL2 ratio under AZ treatment (Figure 4B).

#### 2.3.3. Sildenafil Treatment: WT Mice

Treatment with sildenafil resulted in the normalization of the (mean ± SD) levels of BAX and CD45 in the retina (0.97 ± 0.26 fold and 1.19 ± 1.56 fold, respectively), but marked elevation of TGFβ and TNFα levels (4.42 ± 1.53 fold and 2.91 ± 0.67 fold, respectively). In addition, a decrease in SOD1 levels was detected (0.52 ± 0.18 fold). THY1 levels were elevated (2.8 ± 0.49 fold). STAT3 levels were elevated (2.1 ± 0.77 fold), but this trend was also observed in untreated-model mice (Figure 4A). In the optic nerve, following sildenafil treatment, TGFβ levels decreased (0.69 ± 0.09 fold), BAX and TNFα levels did not change, and CD45 level increased (1.72 ± 0.11 fold). While BAX remained stable, BCL2 expression increased (2.31 ± 0.25 fold), resulting in a decreased BAX/BCL2 ratio of 0.5 (Figure 4B).

#### 2.3.4. Glaucoma Microbead Model: TLR4KO Mice

The levels of CD45 and TNFα were elevated in the retina (4.21 ± 0.38 fold and 1.82 ± 0.56 fold, respectively). However, CD45 expression was still lower than in WT mice (5.45 ± 0.25 fold). BAX was not affected (1.32 ± 1.12 fold and 1.44 ± 0.58 fold, respectively), and the TGFβ level decreased (0.23 ± 0.21 fold) (Figure 4C).

In summary, the molecular analysis revealed that the induction of glaucoma by microbead injection results in an increase in the expression levels of markers of damage (BAX, CD45, and TNFα) in the retina and optic nerve. AZ led to reduced inflammation and apoptosis markers, including BAX and CD45 levels in the retina and BAX/BCL2 ratio in the ON. Treatment with sildenafil resulted in a marked elevation of TNFα, together with a decrease in BAX/BCL2 ratio.

### 2.4. Immunohistochemistry

**Figure 5:**
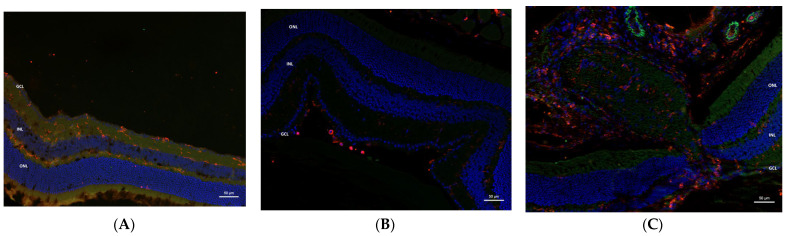
CD45 immunohistochemical staining in AZ-treated eyes: WT mice. (**A**) Eyes with microbead-induced glaucoma and treatment with AZ showed an inflammatory reaction in the inner retinal layers and a milder reaction in the outer plexiform layer. (**B**) No reaction was seen in the internal control left eyes. (**C**) The optic nerve of microbead-injected, AZ-treated eyes also had an intense inflammatory reaction. CD45, red; DAPI, blue; autofluorescence, green. AZ, azithromycin; WT, wild type.

**Figure 6:**
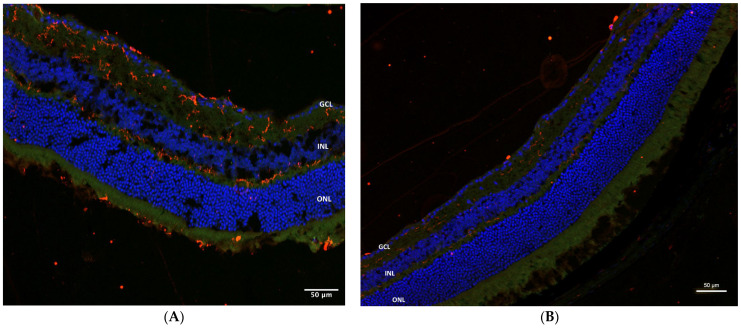
CD45 immunohistochemical staining in AZ-treated eyes: TLR4KO mice. (**A**) Inflammatory reaction is apparent in the retinal layers of the microbead-injected right eyes. (**B**) No staining is seen in the control left eyes. CD45, red; DAPI, blue. AZ, azithromycin; TLR4KO, toll-like receptor 4 knockout.

On CD45 immunohistochemical staining of AZ-treated eyes with microbead-induced glaucoma, an inflammatory reaction was noted in the inner retinal layers and a milder reaction in the outer plexiform layer. The control eyes did not show any staining (Figure 5A,B). The optic nerve also showed an intense inflammatory reaction (Figure 5C). Similar findings were seen in the AZ-treated TLR4KO group (Figure 6A,B).

**Figure 7:**
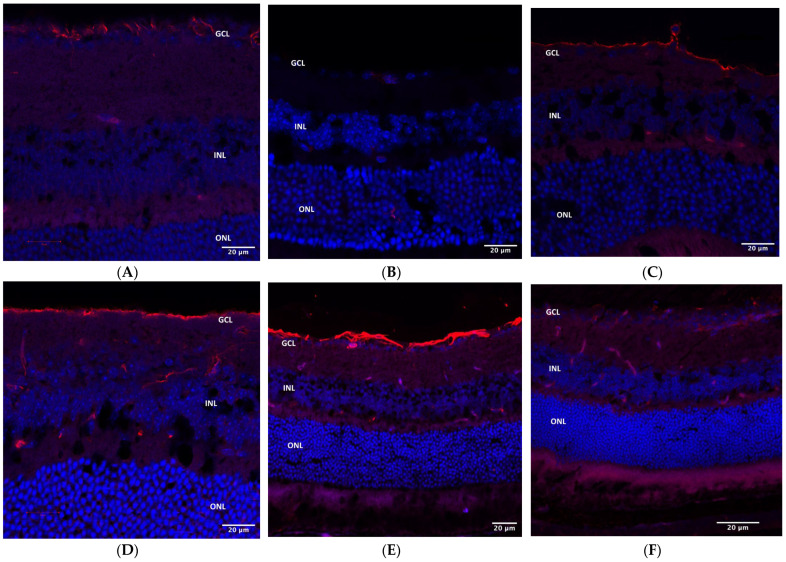
GFAP staining. (**A**) GFAP staining is positive (21.4 RFU) in the right eye of WT microbead-injected eyes. (**B**) GFAP staining is negative (2.79 RFU) in left control eyes. (**C**) WT microbead-injected eyes treated with IP sildenafil showing positive GFAP staining (17.17 RFU). (**D**) Moderate GFAP staining (34.62 RFU) observed in WT microbead-injected eyes treated with AZ. (**E**) TLR4KO microbead-injected eyes treated with AZ showing evident staining (62.685 RFU). (**F**) Minimal staining (14.4 RFU) is seen in TLR4KO left control eyes. GFAP, red; DAPI, blue. RFU, relative fluorescence units; WT, wild type; IP, intraperitoneal; AZ, azithromycin; TLR4KO, toll-like receptor 4 knockout.

In WT mice, GFAP staining was positive in microbead-induced glaucoma eyes without treatment (21.4 relative fluorescence units (RFU)), as well as in eyes treated with intraperitoneal sildenafil (17.17 RFU) or with AZ (34.62 RFU). Control eyes were negative (2.79 RFU) (Figure 7A–D). The TLR4KO mice treated with AZ showed evident staining (62.685 RFU) in the right (microbead-injected) eye (Figure 7E) relative to the left control (14.4 RFU) (Figure 7F).

**Figure 8:**
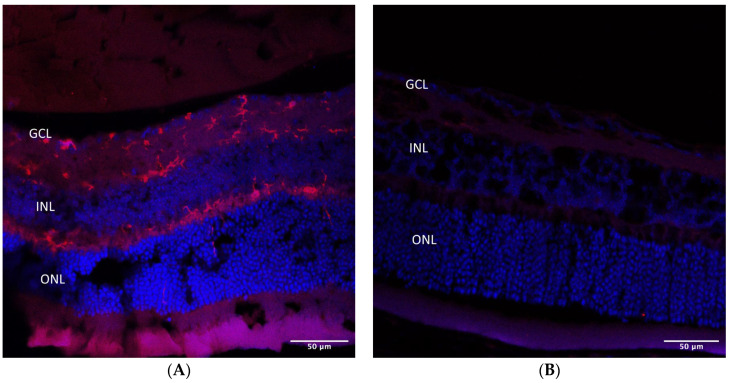
Iba1 staining. (**A**) Iba1 immunohistochemical staining in AZ-treated eyes of WT mice. Microglia activation is evident in the GCL and INL (red). (**B**) Iba1 immunohistochemical staining in control eyes. No microglia activation is noted. Iba1, red; DAPI, blue. AZ, azithromycin; WT, wild type; GCL, ganglion cell layer; INL, inner nuclear layer.

Iba1 staining demonstrated microglia activation in the ganglion cell and inner nuclear layers (GCL and INL) in AZ-treated microbead-injected eyes (Figure 8A) but not in control eyes (Figure 8B).

**Figure 9:**
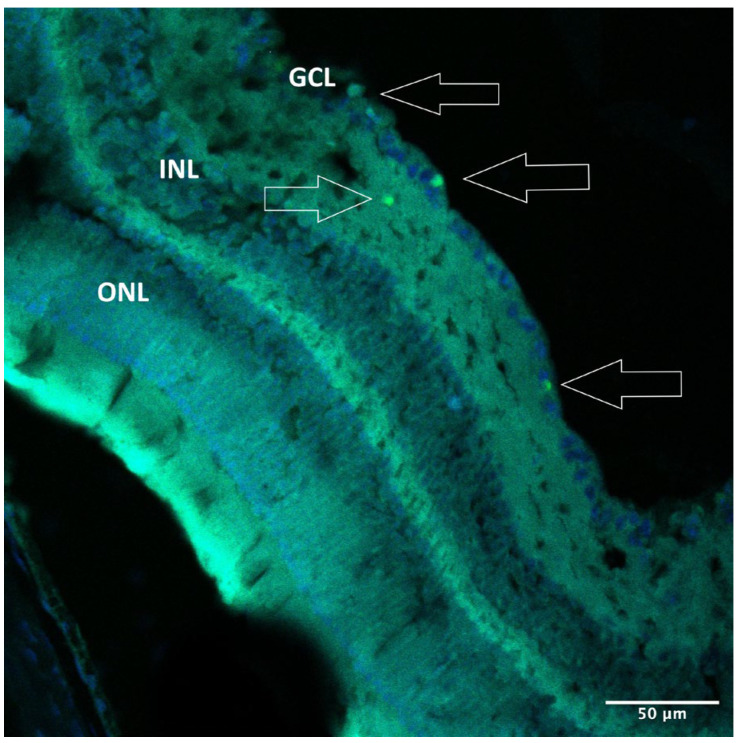
TUNEL immunohistochemical staining in AZ-treated eyes. Few TUNEL-positive apoptotic cells are noted in green in the RGC layer, indicating ganglion cells’ apoptosis death following glaucoma induction (arrows). TUNEL, in situ TdT-mediated dUTP nick end labeling; AZ, azithromycin; RGC, retinal ganglion cell.

In situ TdT-mediated dUTP nick end labeling (TUNEL) assay in AZ-treated microbead-injected eyes showed a number of positive apoptotic cells in the RGC layer, suggesting RGC death following glaucoma induction (Figure 9).

In summary, the immunohistochemistry studies also demonstrated inflammatory and apoptotic response in study eyes (whether with or without medicinal treatment in addition to microbeads) compared with controls. These findings indicate an inflammatory response as part of the glaucomatous process caused by the microbead model.

## 3. Discussion

In the present pilot study, microbead induction of glaucoma in a mouse model was associated with IOP elevation and retinal damage. This effect was less prominent in TLR4KO than WT mice, indicating that the damage was mediated at least in part by inflammation. Treatment with either AZ or sildenafil exerted a neuroprotective effect, although via different routes. Neither drug affected the elevation in IOP.

The higher IOP in eyes with microbead-induced glaucoma than in control eyes on days 3, 7, and 14 of follow-up (Figure 1) is supported by the previous study by our group using the same model, which showed a mild elevation in IOP (mean of 14 mmHg) and significant damage to the RGC layer [13]. Others, however, demonstrated a longer IOP rise in a similar model [19,20,21], possibly due to differences in injection technique or microbead composition. In addition, we observed a wider variability in IOP in the glaucoma model eyes, expressed by a wider standard deviation of the mean IOP on each day of measurement. The wider standard deviation could be the reason that the differences in IOP did not reach statistical significance, especially since IOP was sampled only five times during the follow-up period. Fluctuations in IOP are also a typical finding in human glaucoma, with an increased risk of optic nerve damage [22,23,24].

Treatment with both sildenafil and AZ reduced apoptosis, as shown by histological and molecular studies. Investigation of the drugs’ mechanisms of action in this model suggested that their neuroprotective effect was not mediated by IOP reduction (Figure 2). AZ targeted hypoxia-related genes associated with inflammatory markers (BAX, CD45, TGFβ) and sildenafil increased the TNFα reaction.

On histological analysis, eyes treated with AZ showed an increase in RGC count. In the previous study by our group [13], RGC loss was demonstrated on retinal flat-mount culture in cyan fluorescent protein (CFP) THY1 transgenic mice, as well as by hematoxylin and eosin staining of sections of the eye [13]. Varano et al. [17] investigated the effect of AZ treatment in a rat model of retinal ischemia and reported a statistically significant reduction in RGC loss compared with vehicle-only treatment. A neuroprotective effect of AZ was also reported in several other studies [16,18,25].

The antiapoptotic and neuroprotective effects of sildenafil were also demonstrated by the histological findings. Treatment with sildenafil improved both retinal thickness and RGC count. Although the increase in retinal thickness could also have been due to edema, we presume that the concurrent improvement in RGC count points to a protective effect. Notwithstanding its antiapoptotic effect, sildenafil treatment also led to an increase in other inflammatory markers, suggesting that its protective effect was not mediated by inflammation pathways.

The increase in apoptosis marker (BAX) and inflammatory markers (CD45 and TNFα) in microbead-injected eyes suggests that the model activated apoptosis of the RGC via stress-related and inflammatory mechanisms. This hypothesis was further supported by the statistically significant histological changes. SOD1 levels remained stable after microbead injection, implying that ischemia was not involved.

Similar to our results, in the study of Mac Nair et al. [26] of an optic nerve crush mouse model, TNFα expression levels increased in the study eyes on quantitative polymerase chain reaction analysis (qPCR) relative to the contralateral control eyes. Wang et al. [27] injected hypertonic saline unilaterally into episcleral veins in rats to create an elevated IOP model. Using gene microarray and real-time (RT) qPCR, they investigated the gene expression in RGCs of the glaucomatous eyes. They observed an upregulation of proinflammatory and proapoptotic markers and a downregulation of neuronal prosurvival genes. Like in the present study, albeit in a different glaucoma model, they also observed an increased STAT3 expression level in the glaucomatous eyes.

AZ was given only by the IP route because data on possible intraocular toxicity were lacking. None of the mice showed a toxic effect of IP treatment with AZ. AZ is a commonly used antibiotic with anti-inflammatory properties [16,17,18], and indeed, we observed a reduction in apoptosis and inflammation in the retina (BAX and CD45 levels) and ON (BAX/BCL2 ratio) of AZ-treated microbead-injected eyes.

A recent study reported that microglia secrete TGFβ, which reduces hypoxia-related angiogenesis, resulting in a protective effect [28]. On molecular analysis in the present study, there was a reduction in TGFβ levels in the optic nerve following AZ treatment. We assume that AZ decreased microglia hyperactivity. Interestingly, in microbead-injected eyes, treatment with AZ led to a reduction in CD45 level in the retina, where the microbeads alone led to an increase in CD45, concurrent with its elevation in the optic nerve, where the CD45 levels were unaffected by the microbead injection itself. Additionally, immunostaining with CD45 showed a nonspecific inflammatory reaction in AZ-treated microbead-injected eyes. This also correlates well with the increased microglial activity demonstrated with Iba1 immunostaining. AZ treatment also led to a reduction in TNFα in the optic nerve, further supporting a decrease in microglia activity and reduced inflammation. Our findings are consistent with the study of Ramarao et al. [29], wherein AZ inhibited microglial activation following in vitro white matter injury, leading to a reduced secretion of inflammatory markers, including TNFα, IL-1β, and IL-6 [29].

Sildenafil administration also did not affect IOP (Figure 2). Findings in previous studies on the effect of sildenafil on IOP in animals and humans are controversial, with some showing no change [4] and others reporting a rise in IOP shortly after sildenafil treatment with complete recovery within a few hours [4,30].

Sildenafil was injected via the IP or the IVT route. A previous report from our group in an animal model [7] and several studies in humans [4,10,11] showed that systemic sildenafil administration can induce AION. Therefore, to reduce the risk of optic neuropathy confounding the results, we also evaluated sildenafil administered by the IVT route, which was not associated with toxicity in our previous study [7]. We did not observe AION in the present study. Furthermore, the glaucoma-related damage was even reduced by the sildenafil injection. Accordingly, our previous study in an optic nerve crush model demonstrated that while sildenafil could be nocuous, in the presence of existing optic nerve damage, it was neuroprotective [7]. This paradoxical effect might be associated with the drug’s possible mechanism of action via TNFα, as indicated by the marked elevation in TNFα in the present study. Thus, while in a naïve optic nerve, sildenafil might induce damage, when there is glaucoma-associated neuropathy, the TNF pathway is activated, counterbalancing the glaucoma-induced damage. Mac Nair et al. [26] investigated the role of TNFα in RGC loss in a series of experiments in mice. They observed that intraocular injection of TNFα led to late (after 8 weeks) RGC loss. However, in their optic nerve crush model, when a single TNFα injection was administrated prior to optic nerve crush, RGC loss decreased in the short term, and a worse result was observed in TNFα-knockout mice. The authors concluded that TNFα activation could have a protective effect on RGC.

Our findings on the neuroprotective effect of sildenafil are consistent with previous studies [3,5,31]. The reduction in BAX/BCL2 ratio also supported a preventive effect from apoptosis. Duarte-Silva et al. [31] investigated the effect of sildenafil on experimental autoimmune encephalomyelitis model in mice and found that it modulated the expression of proapoptotic and antiapoptotic molecules, reduced the BAX/BCL2 ratio, and led to better cell survival. The marked elevation in THY1 levels in eyes treated with sildenafil in the present study could suggest activation of ganglion cells. TLR4 is a transmembrane receptor that plays an important role in lipopolysaccharide-mediated immunologic response. Previous studies demonstrated a protective effect in TLR4KO transgenic mice against different oxidative stressogenic disorders, such as myocardial infarction, diabetic retinopathy, and cerebral ischemia [32,33,34]. We previously reported a neuroprotective effect of TLR4KO in an optic nerve crush model, manifested by better RGC survival, reduced CD45 level, and elevated THY1 and BRN3B levels [35]. Chi et al. [36], using an acute ocular hypertension glaucoma model, found histological and molecular evidence of diminished retinal damage and decreased RGC death in TLR4KO mice compared with WT mice.

The TLR4KO mice exhibited a less prominent rise in inflammatory and apoptosis markers than the WT mice, suggesting that in this model, glaucomatous damage is mediated by an inflammatory response.

This pilot study was limited by the small size of some of the study groups. The untreated WT group (8 mice) was too small to compare differences and trends in IOP. However, the effectiveness of microbead-induced glaucoma models in elevating IOP was previously shown [13,19,37,38]. Furthermore, the small size of each individual treatment group resulted in wide confidence intervals such that the results regarding the influence of the different drugs on IOP dynamics and the effect of TLR4KO on this response failed to achieve statistical significance. We believe that further studies in larger samples could clarify the effect of these interventions on IOP and lead to better understanding of glaucoma pathophysiology. Moreover, some of the immunohistochemical studies (CD45, Iba1) were not performed for the microbead-only group (without azithromycin treatment), limiting the ability to evaluate the effect of microbead injection on these markers compared with control eyes beyond the results of the molecular analysis. Another limitation of this study is the lack of protein analysis. Future studies aimed to further investigate the role of TNFα should consider performing protein investigations. In addition, as the statistically significant findings came from the histological analysis, flat-mount retina staining could potentially further support the results, but it was not performed, and should be considered in future studies.

## 4. Materials and Methods

### 4.1. Experimental Animals

The study was conducted in 80 mice, 50 wild-type (WT) C57BL/6 mice and 30 TLR4KO transgenic C57BL/6J mice, aged 6 to 8 weeks with a mean weight of 28 gr. Mice were maintained and handled in accordance with the Association for Research in Vision and Ophthalmology (ARVO) Statement for the Use of Animals in Ophthalmic and Vision Research and the National Institutes of Health guidelines. All animal protocols used in the study were approved by the local Animal Research Committee (b13904_22, b13905_22).

### 4.2. Microbead-Induced Glaucoma Model

To induce glaucoma, a 10% phosphate-buffered saline solution containing 3 µL of urethane microbeads (SUNPU-170, Sunjin Chemical, Gyeonggi-do, Republic of Korea) measuring 17µ in diameter was injected into the anterior chamber of right eyes using a Hamilton^®^ (Reno, NV, USA) syringe with a disposable 30G needle. The injection was performed under anesthesia with ketamine (80 mg/kg) and xylazine (4 mg/kg) (Sigma, St. Louis, MO, USA) supplemented with topical oxybuprocaine hydrochloride 0.4% (Localin, Fischer Pharmaceutical Labs, Tel Aviv, Israel). The microbeads create a sustained increase in IOP by blocking the drainage of the aqueous through the trabecular meshwork. This model was previously established and has been shown to cause IOP elevation, moderate damage, RGC death, and axonal loss [19,37,38,39].

### 4.3. Measurement of Intraocular Pressure

IOP was measured under anesthesia using a rebound tonometer calibrated for mice (Icare^®^ TonoLab, Vantaa, Finland) at baseline (before glaucoma induction) on day 3 and weekly thereafter. Measurements were repeated three times for each reading in each eye, and the average was recorded.

### 4.4. Dosages

Azithromycin (Zithromax^®^, Pfizer, Fareva Amboise, Poce-sur-Cisse, France) was reconstituted in sterile water to 100 mg/mL and then further diluted in saline to reach 10 mg/mL concentration. A dosage of 1 mg in 0.1 mL was injected by the intraperitoneal (IP) route.

Sildenafil citrate (Aurobindo Pharma Limited, Hyderabad, India) dosage was calculated based on previous studies by Walker et al. [40] and our group [7]. In brief, the human dosage used for erectile dysfunction (0.625 to 1.25 mg/kg) and pulmonary hypertension (30 mg per day) were recalculated for mice pharmacokinetics and metabolism. These values were used to calculate the doses of sildenafil for the present study. Sildenafil, 0.24 μg/3 µL, was administered by either a 3 µL intravitreal (IVT) injection into the right eye or a 0.1 mL IP injection.

### 4.5. Treatment with AZ and Sildenafil

AZ was administered IP to 20 WT mice and 9 TLR4KO mice following microbead injection to the right eye. All left eyes were untreated and served as internal controls.

**Figure 10:**
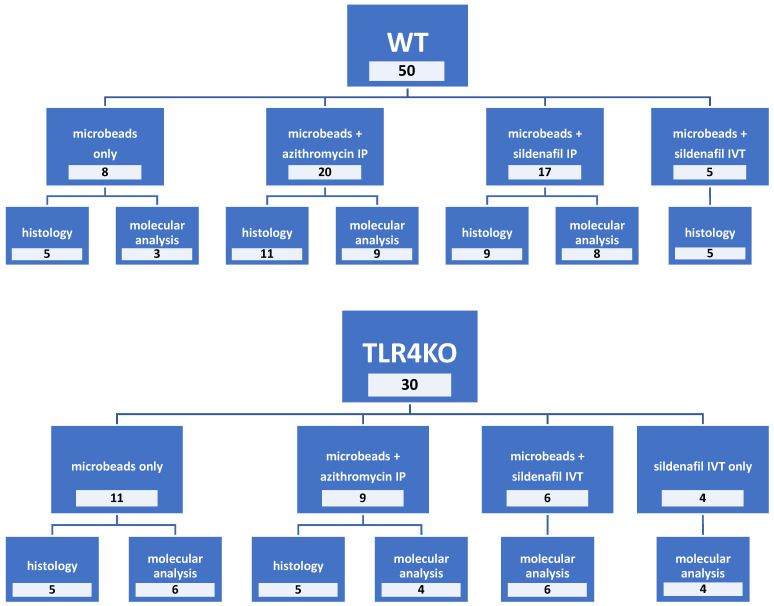
Research group allocation. The figure describes the different procedures and number of mice in each intervention group. Local interventions, including microbead injection, intravitreal azithromycin, or intravitreal sildenafil injections, were given to the right eye only. Both eyes underwent histological or molecular examination. IP, intraperitoneal; IVT, intravitreal; TLR4KO, toll-like receptor 4 knockout; WT, wild type.

Sildenafil was administered IP to 17 WT mice and IVT to 5 WT mice and 6 TLR4KO mice following microbead injection to the right eye. The left eyes were untreated and served as internal controls. In addition, sildenafil was administered to 4 healthy control TLR4KO mice (that were not injected with microbeads). Figure 10 shows the group allocation and treatments.

**Figure 11:**
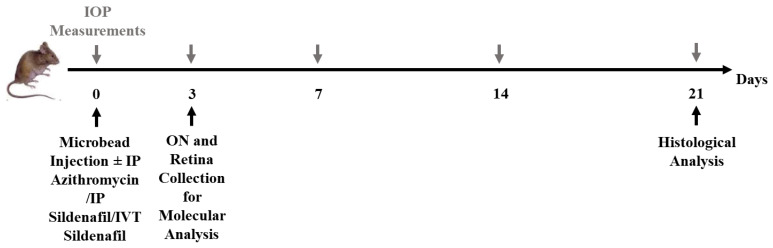
Study design. IOP was measured at baseline, followed by injection of microbeads and administration of treatment according to the treatment group. The IOP was then measured periodically on days 3, 7, 14, and 21. On day 3, mice allocated for molecular analysis were euthanized for retina and optic nerve collection. On day 21, the remaining mice were euthanized for histological analysis. IOP, intraocular pressure, IP, intraperitoneal; IVT, intravitreal; ON, optic nerve.

On day 3, IOP was measured, and nearly half of the WT and TLR4KO groups were euthanized for molecular analysis of the retina and optic nerve. The remaining mice underwent IOP measurements in both eyes on days 7, 14, and 21 and then euthanized for histological examination. Figure 11 schematically summarizes the course of the study.

### 4.6. Molecular Analysis

#### RNA Extraction, Conversion to cDNA, and Analysis by RT-PCR

Molecular analysis was performed on samples obtained on day 3 after glaucoma induction. Retinal tissue and optic nerve were dissected and preserved in RNAlater^®^ solution (Life Science Division, Sigma-Aldrich, St. Louis, MO, USA) at room temperature. RNA was isolated by acid guanidinium thiocyanate–phenol–chloroform extraction using TRIzol^®^ reagent (Life Technologies, Invitrogen, Rhenium Ltd., Modi’in, Israel) according to the manufacturer’s instructions and stored at −80 °C. RNA was reverse-transcribed and amplified in a two-step method. Reverse transcription into complementary deoxyribonucleic acid (cDNA) was performed using random hexamers (Amersham BiosSiences, Buckinghamshire, UK) and Moloney murine leukemia virus reverse transcriptase (Promega, Madison, WI, USA). Amplification was achieved with real-time quantitative PCR (RT-qPCR) using primers of apoptosis-related genes (BAX, BCL2), stress-related genes (SOD1), neuronal/ganglion cell markers (THY1, STAT3), and inflammation-related markers (CD45, TNFα, TGFβ). Reactions were performed in a 10 μL volume containing 1 μL cDNA, 0.5 μM each of the forward and reverse primers, and buffer included in the Master Mix (SYBRR Green I; Applied Biosystems). Gene expression was normalized according to mouse beta actin (ACTB), a housekeeping gene. The primers are listed in Table 2. StepOne Software v2.3 (Applied Biosystems, Foster City, CA, USA) was used for RT-qPCR. Cycling conditions consisted of an initial denaturation step of 95 °C for 10 min, followed by 50 cycles of 1 min at 95 °C and 1 min of annealing and extension at 60 °C. To minimize between-tube variability, duplicate RT-qPCR reactions were performed for each sample, and an average was taken. Threshold cycle efficiency corrections were calculated, and melting curves were obtained using cDNA for each individual-gene PCR assay. The results were quantified by the comparative threshold cycle (Ct) method, where: ΔCt = ΔCt_sample_ − ΔCt_reference gene_ (DataAssist Software v2.2.2, Applied Biosystems).

### 4.7. Histological Analysis

#### 4.7.1. Cryosection

Histological analysis and immunohistochemistry were performed on samples obtained 21 days following glaucoma induction. Enucleated eyes were fixed in 4% formaldehyde for 1 h, washed in phosphate-buffered saline (PBS) (1X; Beit HaEmek, Israel), and then placed in 15% and 20% sucrose dissolved in PBS for 1 h each. Eyes were then placed in 30% sucrose at 4 °C for 12 h, embedded in optimum cutting temperature compound (Sakura Tissue-Tek Tokyo, Japan), and stored at 80 °C for 24 h. Serial cryosections were obtained by cutting the tissues at 10 μm thickness using a Leica CM1850 Cryostat (Leica, Buffalo Grove, IL, USA). Sections were placed on slides at room temperature for 2 h for drying and then stored at −20 °C until staining. Sections were used either for RGC counts and retinal thickness measurement aided by hematoxylin and eosin (H&E) staining or for the identification of the presence of specific proteins by immunofluorescence assays.

#### 4.7.2. H&E Staining, RGC Count, and Retinal Thickness Measurement

Each slide contained 3 consecutive sections that were stained with H&E and examined under a light microscope (Ernst Leitz GmbH, Wetzlar, Germany). Cells were counted in the RGC layer (horizontal counting) in 3 areas (300 µm each) in the midperipheral retina, of every 10 sections of the globe (30 consecutive areas), for a total of 7–10 sections per eye to assess any loss in cell number that may have occurred.

The total retinal thickness was measured in 7–10 H&E-stained sections for each specimen by measuring the distance from the internal limiting layer above the RGC layer to the external limiting membrane. Three measurements per section were performed under a light microscope (Ernst Leitz GmbH, Wetzlar, Germany) with a ×10 objective and scale.

#### 4.7.3. Immunofluorescent Assays

For the immunofluorescent assays, slides were first washed and placed in blocker solution containing 0.5% Triton^TM^ and 5% bovine serum albumin solution for 1 h in order to avoid nonspecific binding. The slides were then incubated at 4 °C overnight with the primary antibodies: anti-CD45 (1:100, RtxMs, Millipore, Temecula, CA, USA), anti-IBA-1 (1:500, Zotal biological instrumentation), and anti-GFAP (1:200, Proteintech, Thermo Fisher Scientific, Waltham, MA, USA); washed in PBS with 0.2% Triton^TM^ X-100 (Sigma-Aldrich, St. Louis, MO, USA); and incubated for 1 h at room temperature with the secondary antibodies: goat anti-rat IgG Alexa Fluor 568 and goat anti-rabbit IgG Alexa Fluor 448 (both 1:200, Molecular Probes, Invitrogen Corporation, Carlsbad, CA, USA). The retinal sections were nuclear-counterstained with Vectashield^®^ antifade mounting medium with 4′,6-diamidino-2-phenylindole (DAPI) (Vector Laboratories, Burlingame, CA, USA). Images were obtained using an Apotome microscope (Zeiss Axio Imager Z2, Oberkochen, Germany).

For the in situ TdT-mediated dUTP nick end labeling (TUNEL) immunostaining, retinal cryosections were examined by TUNEL assay (Roche Diagnostics GmbH, Germany, Cat. No: 11684795910). Staining was performed according to the manufacturer’s instructions. The sections underwent nuclear counterstaining with DAPI. Results were analyzed with a confocal fluorescence microscope (LSM 700 Inverted, Zeiss, Oberkochen, Germany) equipped with appropriate filters. The excitation wavelengths used were 405 nm for DAPI and 488 nm for Cy2.

GFAP immunofluorescence intensity was quantified in the confocal images of mouse retinal sections, as previously described by Haihan Jiao et al. [41]. In brief, sections were analyzed using the NIS-Elements AR software (Ver. 4.50). An average of three locations for each section was used to improve accuracy. Areas were marked with a rectangle from the GCL to the INL. The mean fluorescent intensity for each section was calculated.

### 4.8. Statistical Analysis

Statistical analysis was carried out using SPSS^®^ 26.0 (released 2019, IBM Corp., Armonk, NY, USA). All analyses were two tailed, and the level of significance was set at *p* < 0.05.

RGC counts and retinal thickness measurements on histological sections were compared between groups and analyzed. Right-eye values were compared with left-eye values in each treatment group: microbeads only, microbeads with AZ, and microbeads with sildenafil. Given that these measurements are by nature nonmatched, although they are collected from matched eyes, the independent samples *t*-test was used. In addition, the independent *t*-test was used to compare the effect of AZ or sildenafil versus microbeads only on RGC counts and retinal thickness.

IOP values measured during the study were analyzed. The Wilcoxon test was used to compare IOPs between the right and left eyes (paired samples) at the different time points. The Mann–Whitney U test was used to compare the IOPs of right eyes in WT mice treated with AZ or sildenafil (IP or IVT) to right eyes of mice that received microbeads only. The same calculations were performed in TLR4KO mice. Finally, the IOPs of eyes that received microbeads only (no drugs) were compared between TLR4KO and WT mice in order to evaluate the effect of immunodeficiency on the development of increased IOP in the microbead glaucoma model.

## 5. Conclusions

The microbead glaucoma model is useful for the study of glaucoma pathophysiology and for investigating new potential drugs. This study showed that glaucomatous damage was largely mediated by inflammatory processes and apoptosis. The findings did not support an ischemic reaction. Treatment with AZ diminished the inflammatory reaction expressed histologically and molecularly and resulted in an improvement in RGC count in eyes with microbead-induced glaucoma. Sildenafil acted via TNFα pathways and had a neuroprotective effect, also resulting in the preservation of RGCs.

## Figures and Tables

**Table 1:** (**A**) Comparison of retinal thickness and RGC count in WT mice within treatment groups: right eyes with microbead-induced glaucoma under conditions of no treatment or AZ or sildenafil treatment compared with left control eyes. (**B**) Comparison of retinal thickness and RGC counts in WT mice between treatment groups: right eyes with microbead-induced glaucoma, untreated versus treated with either AZ or sildenafil.

(A)					
Group	Eye	Retinal Thickness (µm)		Retinal Ganglion Cell Count (Number of Cells in 200 µm Section)	
		Mean (SD)	*p*-Value	Mean (SD)	*p*-Value
Microbeads only	right	183.27 (30.93)	0.009	20.37 (3.82)	0.003
	left	198.34 (28.94)		22.41 (4.35)	
Microbeads + IP azithromycin	right	189.72 (23.71)	0.002	21.87 (4.92)	<0.001
	left	200.79 (27.98)		25.13 (5.02)	
Microbeads + IP or IVT sildenafil	right	192.55 (17.68)	0.565	21.12 (4.71)	0.798
	left	193.79 (19.42)		21.24 (3.9)	
Microbeads + IP sildenafil	right	193.44 (17.21)	0.363	20.68 (4.56)	0.145
	left	195.73 (18.91)		21.42 (3.67)	
Microbeads + IVT sildenafil	right	186.99 (19.97)	0.405	23.96 (4.8)	0.003
	left	191.3 (19.92)		21.06 (4.12)	
**(B)**					
**Group**		**Retinal Thickness (** **µm)**		**Retinal Ganglion Cell Count (Number of Cells in 200** **µm Section)**	
		**Mean (SD)**	*p*-Value *	**Mean (SD)**	*p*-Value *
Microbeads only		183.27 (30.93)		20.37 (3.82)	
Microbeads + IP azithromycin		189.62 (23.71)	0.188	21.87 (4.92)	0.017
Microbeads + IP or IVT sildenafil		192.55 (17.68)	0.037	21.12 [4.71)	0.226

* *p*-Value for mean difference from microbeads-only group. IP, intraperitoneal; IVT, intravitreal.

**Table 2:** List of primers used for the molecular analysis.

Marker	Primer Code
ACTB_F	TAGGCACCAGGGTGTGATGGT
ACTB_R	CATGTCGTCCCAGTTGGTAACA
BAX_F	CTGAGCTGACCTTGGAGC
BAX_R	GACTCCAGCCACAAAGATG
BAX_2_F	AGGATGCGTCCACCAAG
BAX_2_R	AAGTAGAAGAGGGCAACCAC
BCL-2_F	CCTGTGGATGACTGAGTACCT
BCL-2_R	GAGCAGGGTCTTCAGAGACA
BCL-2_F_2	GTGGGGCGGGAGTCGGGACT
BCL-2_R_2	GACCCAGAATCCACTCACAC
CD45_F	GAACATGCTGCCAATGG
CD45_R	TGTCCCACATGACTCCTT
SOD-1_F	GCCCGGCGGATGAAGA
SOD-1_R	CGTCCTTTCCAGCAGTCACA
STAT3_F	TTATCAGCTTAAAATTAAAGTGTGC
STAT3_R	ATTCCCACATCTCTGCTCCC
TGFb_F	ATGACATGAACCGGCCC
TGFb_R	ACTTCCAACCCAGGTCC
THY1_F	ACATGTGTGAACTTCGAGTCTCGGG
THY1_R	GCTTATGCCACCACACTTGACCAG
TNFα_F	TCTCAAAATTCGAGTGACAAGC
TNFα_R	ACTCCAGCTGCTCCTCCAC

## Data Availability

Data is contained within the article.
